# Factors associated with musculoskeletal pain in the spinal column of children and adolescents: a cross-sectional study with network analysis

**DOI:** 10.1590/1984-0462/2026/44/2026040

**Published:** 2026-09-18

**Authors:** Marcella Veronnica Pereira Gomes, Ruth Stefany Monteiro Belém, Amanda Vitória Gomes Pantoja, Beatriz dos Santos Costa, Naina Yuki Vieira Jardim, Alex Harley Crisp, Maurício Oliveira Magalhães

**Affiliations:** a Universidade Federal do Pará, Belém, PA, Brazil.; b Universidade Estadual do Pará, Tucuruí, PA, Brazil.

**Keywords:** Spine, Pain, Child, Adolescent, Musculoskeletal pain, Coluna vertebral, Dor, Criança, Adolescente, Dor musculoesquelética

## Abstract

**Objective:**

The aim of this study was to analyze the relationship between musculoskeletal spinal pain and individual, behavioral, and contextual factors in children and adolescents.

**Methods:**

A cross-sectional study was conducted with 189 children and adolescents (10–16 years), of both genders, enrolled in public and private schools in Belém, Pará, Brazil. Musculoskeletal pain and postural habits were assessed using the Back Pain and Posture Evaluation Instrument for Children and Adolescents, a validated questionnaire for this population. Network analysis using the EBICglasso estimator explored associations between individual (age, gender), behavioral (physical activity in school and leisure contexts), and contextual factors (school type and postural habits related to daily activities and electronic device use) and the presence of neck and low back pain.

**Results:**

The prevalence of low back pain was 62.6% and neck pain 65.4%. Low back pain was negatively associated with family history of pain (r=-0.14) and positively associated with female gender (r=0.19) and neck pain (r=0.12). Sitting posture during electronic device use was negatively associated with neck pain (r=-0.19). Gender and age showed higher centrality, indicating greater influence within the network, while physical activity showed a negative influence on pain outcomes.

**Conclusions:**

Musculoskeletal spinal pain was highly prevalent and associated with multiple interrelated factors. Network analysis highlights its multifactorial nature and provides relevant insights into key variables that may guide integrated health promotion and early prevention strategies in pediatric populations.

## RESUMO

**Objetivo:**

Analisar a relação entre dor musculoesquelética na coluna vertebral e fatores individuais, comportamentais e contextuais em crianças e adolescentes.

**Métodos:**

Estudo transversal com 189 crianças e adolescentes (10–16 anos), de ambos os gêneros, matriculados em escolas públicas e privadas de Belém, Pará, Brasil. A dor musculoesquelética e os hábitos posturais foram avaliados por meio do Instrumento de Avaliação de Dor nas Costas e Postura Corporal para Crianças e Adolescentes (BackPEI-CA). A análise de rede foi conduzida com o estimador EBICglasso para explorar associações entre fatores individuais (idade e sexo), comportamentais (atividade física) e contextuais (tipo de escola e hábitos posturais) e a presença de dor cervical e lombar.

**Resultados:**

A prevalência de dor lombar foi de 62,6% e de dor cervical, 65,4%. A dor lombar apresentou associação negativa com histórico familiar de dor (r=-0,14) e positiva com o gênero feminino (r=0,19) e com dor cervical (r=0,12). A postura sentada ao utilizar dispositivos eletrônicos apresentou associação negativa com dor cervical (r=-0,19). Sexo e idade apresentaram maiores medidas de centralidade na rede, enquanto a prática de atividade física apresentou influência negativa.

**Conclusões:**

A dor musculoesquelética na coluna vertebral apresentou elevada prevalência e esteve associada a múltiplos fatores inter-relacionados. A análise de rede reforça sua natureza multifatorial e destaca a importância de estratégias integradas de promoção da saúde e prevenção precoce.

## INTRODUCTION

Musculoskeletal pain is a common condition in children and adolescents, with an estimated prevalence between 4% and 40%, representing an important public health problem in this age group. Data from the Global Burden of Disease Study indicate that cervical and lumbar spine pain are among the leading causes of years lived with disability (YLDs) in individuals aged 10–19 years, affecting approximately 2.4 million people worldwide. In addition to the immediate functional impact, this type of pain is associated with a higher risk of symptom persistence and chronicity in adulthood, generating direct and indirect costs for health systems.^1–3^

Understanding the factors associated with musculoskeletal pain in childhood and adolescence is essential, as this period represents a critical phase of physical, behavioral, and psychosocial development. Recent studies have highlighted that poor posture habits, sedentary behavior, prolonged use of electronic devices, and low levels of physical activity are often associated with the occurrence of spinal pain in schoolchildren, reinforcing the need for investigations that integrate multiple risk factors.^4–6^

Postural habits comprise the positions and movements adopted during activities of daily living, such as sitting, writing, using electronic devices, carrying school backpacks, and sleeping. Repeated exposure to poor posture during the school period can contribute to mechanical overload of the spine, favoring the onset of musculoskeletal pain. In this context, the Back Pain and Posture Evaluation Instrument (BackPEI), validated for Brazilian children and adolescents (BackPEI-CA), has been widely used to investigate the presence of back pain and its associated factors, including postural, behavioral, and demographic aspects.^7–9^

Despite the growing number of studies analyzing factors associated with musculoskeletal pain in children and adolescents, most investigations use traditional analytical approaches, focused on the isolated assessment of sociodemographic, behavioral, or postural variables.^4,8^ In contrast, more recent methodological approaches, such as network analysis, have been proposed to better capture the complex interrelationships among multiple factors.^10,11^ Additionally, studies in this field are commonly conducted and reported in accordance with established methodological guidelines.^12^ In this context, there are still a few studies that explore the simultaneous interaction between multiple individual, behavioral, and contextual factors, which may limit the understanding of the complexity involved in the development of musculoskeletal pain in this population.^13–15^

Network analysis has emerged as a promising statistical approach in the field of health, as it allows for the investigation of interrelationships between variables in an integrated manner, identifying direct and indirect connections between risk factors and clinical outcomes. Although still under-explored in pediatric epidemiological studies, this approach can contribute to a more comprehensive understanding of the patterns of association between musculoskeletal pain, postural habits, and individual characteristics in children and adolescents.^10,11^

Therefore, the present study aimed to analyze the relationship between musculoskeletal pain in the spine and individual, behavioral, and contextual factors in children and adolescents.

## METHOD

This cross-sectional, observational, and exploratory study was conducted with children and adolescents enrolled in public and private schools in the city of Belém, Pará, Brazil, with the aim of describing the occurrence of musculoskeletal pain in the spine and exploring associations between individual, behavioral, and contextual factors in a pediatric population. The study was approved by the Research Ethics Committee of the Federal University of Pará (CAAE: 23163519.6.0000.0018), and all participants and their legal guardians were informed about the objectives and procedures of the research, with anonymity and confidentiality of information guaranteed, in accordance with the ethical principles of the Declaration of Helsinki. The reporting of this study followed the Strengthening the Reporting of Observational Studies in Epidemiology (STROBE) guidelines.^12^

The study population consisted of children and adolescents of both sexes, aged between 10 and 16 years, who were regularly enrolled in elementary and high school. Participants were recruited from public and private schools that were invited to participate in the study. A total of 20 public schools were invited, of which five agreed to participate, and five private schools were invited, of which three agreed to participate. Children and adolescents who presented the Free and Informed Consent Form signed by their legal guardians, as well as the Assent Form, when applicable, were included, regardless of the presence of musculoskeletal pain. Participants with a difference in lower limb length greater than or equal to 1.5 cm, assessed using the apparent discrepancy test, measuring the distance between the umbilical scar and the medial malleolus, were excluded. Those with a history of orthopedic, neurological, neuromuscular, or cardiorespiratory diseases, previous orthopedic surgery, or developmental disorders that could interfere with the evaluation were also excluded.

Due to the exploratory nature of the study and the analytical approach based on network analysis, all students who met the eligibility criteria and agreed to participate during the data collection period were included. The final sample consisted of 189 participants.

After authorization from the Pará State Department of Education, the project was presented to the principals of the participating schools. In private schools, educational lectures on posture and back pain were held, followed by the delivery of the Free and Informed Consent Form to legal guardians. Data collection was carried out on the premises of the schools themselves, in a reserved space, by a team composed of physical therapists and previously trained physical therapy students, after the return of the duly signed consent form.

Participants completed an assessment form containing sociodemographic and clinical information, including gender, age, manual dominance, and physical activity. Family history of musculoskeletal pain was assessed through a self-reported dichotomous question (yes/no), based on the participant's report of similar pain in family members. This information was collected using an electronic form (Google Forms) to standardize records and reduce transcription errors. Body mass was measured with a digital scale, and height was measured using a tape measure fixed to the wall, with the participant barefoot and in an upright position. Body mass index was calculated as the ratio of body mass in kilograms to height in square meters.

Musculoskeletal pain and postural habits were assessed using the BackPEI-CA, consisting of 30 multiple-choice questions. Questions 1–20 assess postural and behavioral risk factors, including sitting posture during school activities, posture when using electronic devices (cell phones, tablets, and computers), posture when picking up objects from the floor, sleeping position, and the method of carrying school backpacks, while questions 21–30 investigate the presence, frequency, and intensity of back pain. The frequency of low back and neck pain was assessed using specific items of the instrument that capture the presence and recurrence of symptoms according to predefined response categories. Pain intensity was measured using the Numeric Rating Scale (NRS), ranging from 0 (no pain) to 10 (worst possible pain). Postural habits were classified according to the predefined categories of the instrument, which include illustrated options representing different body positions during daily activities. Participants were instructed to select the option that best represented their usual posture. For analytical purposes, these responses were categorized as adequate or inadequate according to the criteria established by the instrument. The instrument also assesses the impact of pain on school attendance and participation in recreational activities, using gender-specific versions, as recommended by the authors of the questionnaire.

Initially, a descriptive analysis of the data was performed, presenting means, standard deviations (SDs), medians, and absolute and relative frequencies, according to the nature of the variables. Categorical variables were presented as absolute and relative frequencies, whereas continuous variables were summarized using means and SDs. The main outcomes of the study were the presence of low back pain and neck pain, assessed using the BackPEI-CA. Independent variables included sociodemographic characteristics (age, gender, and type of school), behavioral factors (physical activity in different contexts), and postural habits related to daily activities (e.g., sitting posture at school, posture when picking up objects, and sleeping position) and the use of electronic devices (e.g., sitting and standing posture when using cell phones, tablets, and computers). To explore the associations between individual and behavioral factors and the presence of musculoskeletal pain in the spine, network analysis was applied. The EBICglasso estimator was used, which combines LASSO (Least Absolute Shrinkage and Selection Operator) graphical regularization with the Extended Bayesian Information Criterion, allowing the identification of a parsimonious network, maintaining only the most relevant associations between variables. A penalty parameter of 0.25 was adopted for LASSO regularization. The network visualization was generated using the spring layout algorithm, in which the proximity between nodes represents the strength of the associations. Blue edges indicate positive associations, while red edges indicate negative associations, with the thickness of the lines proportional to the magnitude of these associations. The relative importance of each variable in the network was assessed using the metrics of betweenness, closeness, strength, and expected influence. All analyses were performed using JASP software, version 0.18.3.

## RESULT

A total of 189 students aged 10–16 years from public and private schools were included in the study. Schools were invited to participate, and a total of five public and three private schools agreed to participate. Participants were recruited according to predefined eligibility criteria, and those who did not meet the inclusion criteria or met exclusion criteria were not included in the final sample. Of the total sample, 76.7% were from public schools. Of the total sample, 48.6% were female, with a mean age of 11.5 years (SD=1.6). The sociodemographic and clinical characteristics of the participants are presented in Table 1.

**Table 1:** Sociodemographic and clinical characteristics of schoolchildren participating in the study (n=189).

Characteristics		n=189	Female (n=94)	Male (n=95)
Age (years), mean (SD)		11.5 (1.6)	11.8 (1.6)	11.2 (1.6)
Weight (kg), mean (SD)			46.5 (13.8)	44.3 (13.1)
Height (m), mean (SD)			1.49 (0.1)	1.48 (0.1)
BMI (kg/m^2^), mean (SD)			20.5 (5.4)	20.5 (5.4)
Educational Institutions				
	Public institution	145 (76.7%)	-	-
	Private institution	44 (23.2%)	-	-
Low back pain, n (%)		118 (62.6)	-	-
Neck pain, n (%)		124 (65.4)	-	-
Intensity of low back pain, mean (NRS)		4.6	-	-
Neck pain, n (%)		124 (65.4)	-	-

SD: standard deviation; BMI: body mass index; NRS: Numeric Rating Scale.

Source: Prepared by the authors.

Regarding musculoskeletal pain in the spine, a high prevalence of both low back and neck pain was observed among participants. Pain intensity was moderate for both regions, as assessed by the Numeric Rating Scale (Table 1).

Regarding postural and behavioral habits, a high prevalence of inadequate postures was observed across several daily activities, particularly during sitting and object-handling tasks. Sleep patterns were generally within recommended ranges, with the lateral decubitus position being the most frequently reported (Tables 2 and 3). Regarding the use of electronic devices, a considerable proportion of participants reported prolonged use, particularly of mobile phones, while computer use was less frequent (Table 2).

**Table 2:** Distribution of behavioral habits of schoolchildren according to gender.

Variable (n)		Male n (%)	Female n (%)
Time spent watching television per day (n=189; hours)			
	0–3	62 (65.3)	61 (64.9)
	4–7	12 (12.6)	9 (9.6)
	≥8	2 (2.1)	1 (1.1)
	Don't know how to answer, or depends on the day	19 (20.0)	23 (24.5)
Time spent using the computer per day (hours)			
	0–3	46 (48.4)	51 (54.3)
	4–5	3 (3.2)	1 (1.1)
	≥6	3 (3.2)	3 (3.2)
	Don't know how to answer, or depends on the day	43 (45.3)	39 (41.5)
Mobile phone usage time (hours)			
	0–3	46 (48.4)	39 (41.5)
	4–5	18 (18.9)	15 (16.0)
	≥6	17 (17.9)	25 (26.6)
	Don't know how to answer, or depends on the day	14 (14.7)	15 (16.0)
Reading, studying, and/or using a cell phone/tablet in bed			
	No	54 (56.8)	64 (68.1)
	Sometimes	14 (14.7)	10 (10.6)
	Yes	27 (28.4)	20 (21.3)
Sleeping position			
	Supine	7 (7.4)	14 (14.9)
	Lateral decubitus	66 (69.5)	59 (62.8)
	Prone position	16 (16.8)	16 (17.0)
	Don't know how to answer, or depends on the day	6 (6.3)	5 (5.3)
Sleep time per night (hours)			
	0–7	31 (32.6)	41 (43.6)
	8–9 (Adequate)	42 (44.2)	38 (40.4)
	≥10	18 (18.9)	13 (13.8)
	Don't know how to answer, or depends on the day	4 (4.2)	2 (2.1)

Percentages were calculated separately for males (n=95) and females (n=94).

Source: Prepared by the authors.

**Table 3:** Distribution of postural habits of schoolchildren according to gender.

Variable (n)		Male n (%)	Female n (%)
Sitting posture for writing			
	Adequate	9 (9.5)	17 (18.1)
	Inadequate	83 (87.4)	77 (81.9)
	Don't know or other	3 (3.2)	-
Sitting posture on a chair and/or bench			
	Adequate	18 (18.9)	20 (21.3)
	Inadequate	73 (76.8)	74 (78.7)
	Don't know or other	4 (4.2)	-
Sitting posture when using the computer			
	Adequate	6 (6.3)	11 (11.7)
	Inadequate	38 (40.0)	37 (39.4)
	Don't know or other	51 (53.7)	46 (48.9)
Sitting posture when using a cell phone/tablet			
	Adequate	10 (10.5)	12 (12.8)
	Inadequate	78 (82.1)	76 (80.9)
	Don't know or other	7 (7.4)	6 (6.4)
Standing posture when using a cell phone/tablet			
	Adequate	29 (30.5)	13 (13.8)
	Inadequate	62 (65.3)	74 (78.7)
	Don't know or other	4 (4.2)	7 (7.4)
Posture for picking up objects from the floor			
	Adequate	5 (5.3)	17 (18.1)
	Inadequate	87 (91.6)	76 (80.9)
	Don't know or other	3 (3.2)	1 (1.1)
Means of transporting school supplies			
	Two-strap school backpack	91 (95.8)	88 (93.6)
	Other means of transport (briefcase, bag, etc.)	4 (4.2)	6 (6.4)
Method of carrying a school backpack			
	Adequate (symmetrical shoulder straps)	81 (85.3)	71 (75.5)
	Inadequate (asymmetrical mode)	12 (12.6)	12 (12.6)

Percentages were calculated separately for males (n=95) and females (n=94).

Source: Prepared by the authors.

**Figure 1:**
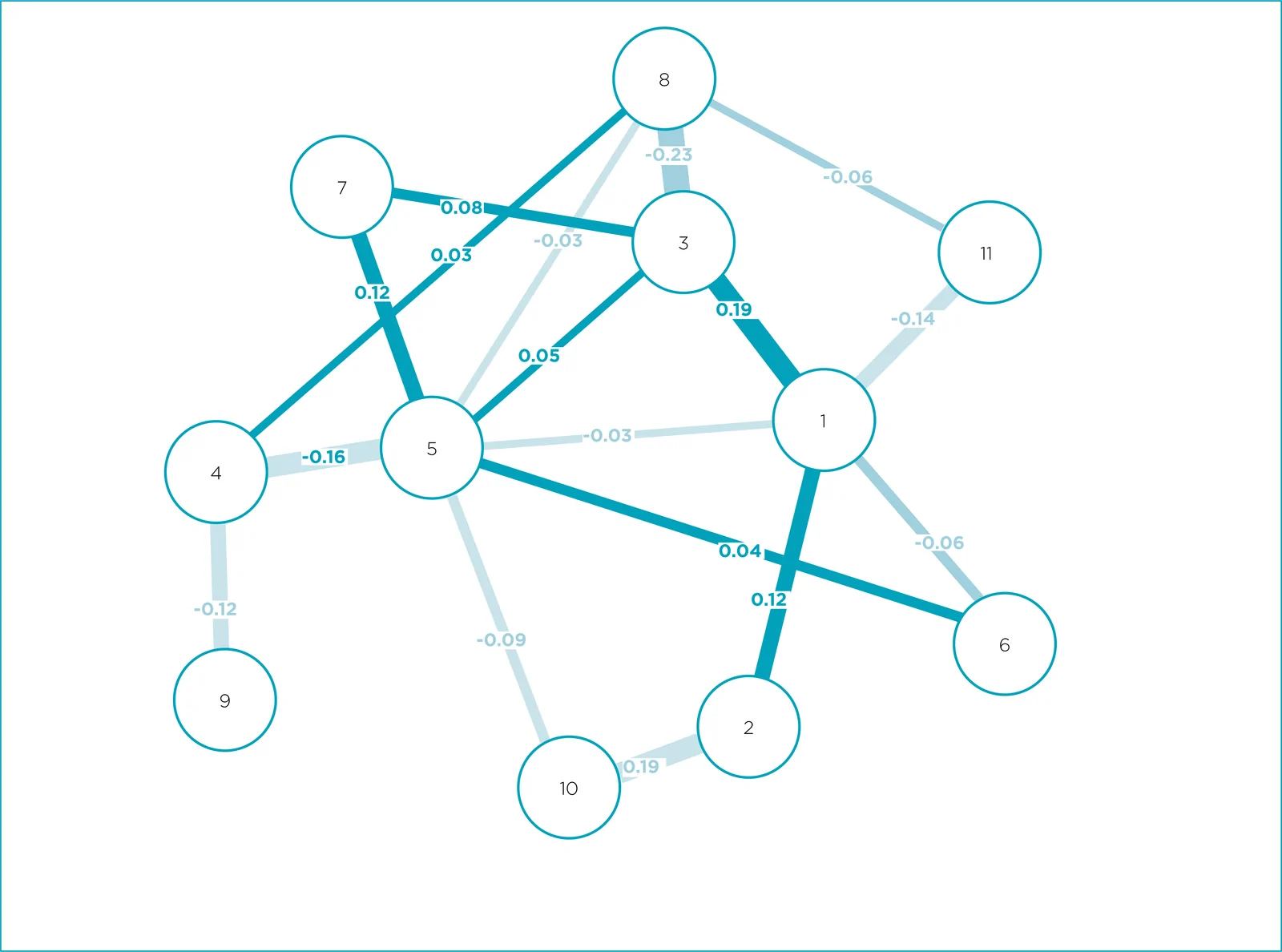
Network of associations between variables reported by students.

Source: Prepared by the authors.

Network analysis revealed associations between individual and behavioral factors and the presence of musculoskeletal pain in the spine (Figure 1). Low back pain was negatively correlated with family history of pain (r=-0.14) and positively correlated with female gender (r=0.19) and the presence of neck pain (r=0.12). The position when sitting to use a cell phone or tablet showed a negative correlation with neck pain (r=-0.19). In addition, a negative correlation was observed between gender and physical activity outside school.

1: Low back pain; 2: Neck pain; 3: Gender; 4: School administrative sphere; 5: Age; 6: Body mass index; 7: Physical activity at school; 8: Physical activity outside school; 9: Position when sitting at school; 10: Position when sitting while using a cell phone/tablet; 11: Family history of pain.

The centrality metrics indicated that the variables age (betweenness=1420) and gender (betweenness=1278) had the highest intermediation values in the network, suggesting greater influence on the flow of connections between the other variables. The gender variable also had the highest values for closeness (closeness=1301) and strength (strength=1450). Regarding expected influence, physical activity at school had a negative value (expected influence=-1764), as did the position when sitting to use a cell phone or tablet (expected influence=-1144). The centrality and expected influence metrics are shown in Table 4.

**Table 4:** Centrality and expected influence metrics of the variables included in the network analysis.

Variable	Betweenness	Closeness	Strength	Expected influence
Low back pain	1.562	1.093	1.397	1.004
Neck pain	-0.142	0.676	-0.087	0.098
Gender	1.278	1.301	1.450	1.150
Type of school	0.426	-0.542	0.004	-0.999
Age	1.420	0.667	1.240	-0.111
BMI	-0.852	-1.394	-1.332	0.389
Physical activity in school	-0.852	0.205	-0.753	1.764
Physical activity outside school	-0.852	0.003	0.252	-1.247
School sitting position	-0.852	-1.773	-1.190	-0.214
Sitting position while using a cell phone/tablet	-0.284	0.499	-0.264	-1.144
Family pain history	-0.852	-0.735	-0.716	-0.690

Positive values indicate greater centrality or positive influence in the network; negative values indicate negative influence.

Source: Prepared by the authors.

## DISCUSSION

This study analyzed the relationship between musculoskeletal pain in the spine and individual, behavioral, and contextual factors in children and adolescents using an exploratory approach based on network analysis. The findings showed a high prevalence of low back and neck pain, as well as associations between musculoskeletal pain, gender, age, physical activity, and postural habits, reinforcing the multifactorial nature of this outcome in the pediatric population.

The prevalences of low back pain (62.6%) and neck pain (65.4%) observed are consistent with national and international studies that point to musculoskeletal pain as frequent during childhood and adolescence, with the potential to persist over time.^13–16^ These findings reinforce the relevance of musculoskeletal pain as a public health problem already at school age. Several factors may help explain these estimates. First, the age range evaluated (10–16 years) corresponds to a developmental stage in which musculoskeletal symptoms tend to become more frequent due to rapid growth, behavioral changes, and increased exposure to sedentary activities.^13–18^ Second, the BackPEI-CA is a validated self-report instrument that captures both current and recurrent episodes of spinal pain, which may contribute to higher prevalence estimates compared with studies using stricter definitions of pain.^7^ In addition, contextual factors related to lifestyle, such as prolonged use of electronic devices and the high prevalence of inadequate postural habits observed in the present sample, may also contribute to the occurrence of musculoskeletal pain in this population.^19–24^

Gender was central to the network and positively associated with low back pain, consistent with evidence indicating a higher prevalence of musculoskeletal pain among girls, especially during adolescence.^13–16^ Age also showed high centrality, corroborating studies that identify adolescence as a critical period for the emergence and consolidation of musculoskeletal symptoms, possibly due to bodily and behavioral changes and increased time spent in sedentary activities.^13–16^

Physical activity, particularly in the school environment, had a negative influence on the network, suggesting an association with less impact from musculoskeletal pain. Although network analysis does not allow causal inferences, this finding is in line with international guidelines and systematic reviews that indicate regular physical activity as a potentially protective factor for the musculoskeletal health of children and adolescents.^18^

A high prevalence of poor posture habits was observed during school activities and when using electronic devices. The posture adopted when sitting to use a cell phone or tablet was associated with neck pain, suggesting that poor posture behaviors may be related to neck pain. However, the literature presents inconsistent results regarding the direct association between the use of electronic devices and musculoskeletal pain, indicating that factors such as sleep quality, physical inactivity, and biopsychosocial aspects may play a more relevant role.^19–24^ In addition, factors such as sleep patterns and ergonomic conditions may also influence musculoskeletal health in children and adolescents.^25–27^ Other ergonomic factors commonly discussed in the literature include the transport of school backpacks, which has been investigated as a potential contributor to back pain in schoolchildren, although current evidence remains inconclusive.^28,29^

In the present study, a considerable portion of participants reported sleep duration close to the recommended amount. Recent evidence points to a bidirectional relationship between sleep disorders and chronic musculoskeletal pain, indicating that sleep problems may increase the risk of pain in both the short and long term.^25,26^ In addition, inadequate ergonomic conditions in the school environment, such as furniture that is not adapted to anthropometric characteristics, can contribute to musculoskeletal overload.^27^

The use of network analysis represents a methodological advance, as it allows the integrated exploration of the interrelationships between multiple factors associated with musculoskeletal pain, identifying central variables that might not be evident in traditional analytical approaches. However, some limitations should be considered when interpreting the results. First, the cross-sectional design does not allow causal inferences. Second, the use of self-reported measures may introduce recall bias. In addition, the findings of this study should be interpreted considering some limitations. Although invited schools agreed to participate to varying extents, the sample was obtained through a non-probabilistic strategy, which may limit its representativeness, particularly regarding socioeconomic diversity. In addition, the restriction to a single Brazilian city may affect the generalizability of the findings. Despite these limitations, the study included students from both public and private schools, providing a heterogeneous sample of schoolchildren. Future multicenter studies with probabilistic sampling and longitudinal designs are recommended to confirm and expand these findings in broader populations.

In conclusion, this study showed a high prevalence of musculoskeletal pain in the spine in children and adolescents, as well as associations with individual, behavioral, and contextual factors. The network analysis highlighted gender, age, physical activity, and postural habits as central variables in the observed interrelationships. These findings reinforce the multifactorial nature of musculoskeletal pain in the pediatric population and the importance of integrated approaches aimed at health promotion and early prevention.

## Data Availability

The dataset that supported the findings of this study is available from the corresponding author upon reasonable request.

## References

[1] King S, Chambers CT, Huguet A, MacNevin RC, McGrath PJ, Parker L (2011). The epidemiology of chronic pain in children and adolescents revisited: a systematic review. Pain.

[2] Vitta A, Bento TP, Cornelio GP, Perrucini PD, Felippe LA, Conti MH (2021). Incidence and factors associated with low back pain in adolescents: a prospective study. Braz J Phys Ther.

[3] Global Burden of Disease Pediatrics Collaboration (2016). Global and national burden of diseases and injuries among children and adolescents between 1990 and 2013: findings from the Global Burden of Disease 2013 Study. JAMA Pediatr.

[4] Kamper SJ, Henschke N, Hestbaek L, Dunn KM, Williams CM (2016). Musculoskeletal pain in children and adolescents. Braz J Phys Ther.

[5] Wang R, Yin Y, Zhang H, Pan L, Zhu Y, Wang M (2023). Risk factors associated with the prevalence of neck and shoulder pain among high school students: a cross-sectional survey in China. BMC Musculoskelet Disord.

[6] Yang S, Werner BC, Singla A, Abel MF (2017). Low back pain in adolescents: a 1-year analysis of eventual diagnoses. J Pediatr Orthop.

[7] Rosa BN, Candotti CT, Pivotto LR, Noll M, Silva MG, Vieira A (2022). Back pain and body posture evaluation instrument for children and adolescents (BackPEI-CA): expansion, content validation, and reliability. Int J Environ Res Public Health.

[8] Noll M, Candotti CT, Tiggemann CL, Schoenell MC, Vieira A (2013). Prevalência de hábitos posturais inadequados de escolares do ensino fundamental da cidade de Teutônia: um estudo de base populacional. Rev Bras Ciênc Esporte.

[9] Dantas MG, Aquino AN, Correia HJ, Ferreira KP, Nascimento BB, Silva LD (2021). Prevalence of back pain and idiopathic scoliosis in adolescents from the semiarid region of Brazil: a cross-sectional study. J Chiropr Med.

[10] Marteleto RM (2001). Análise de redes sociais: aplicação nos estudos de transferência da informação. Cienc Inf.

[11] Leme DE, Alves EV, Lemos VC, Fattori A (2020). Network analysis: a multivariate statistical approach for health science research. Geriatr Gerontol Aging.

[12] Sharp MK, Bertizzolo L, Rius R, Wager E, Gómez G, Hren D (2019). Using the STROBE statement: survey findings emphasized the role of journals in enforcing reporting guidelines. J Clin Epidemiol.

[13] Hatakeyama BA, Camargo BI, Santos VS, Leite MN, Santo CM, Kamper SJ (2024). Prevalence of disabling musculoskeletal pain in children and adolescents in Brazil: a cross-sectional study. Braz J Phys Ther.

[14] Noll M, Candotti CT, Rosa BN, Vieira A, Loss JF (2021). Back pain and its risk factors in Brazilian adolescents: a longitudinal study. Br J Pain.

[15] Chambers CT, Dol J, Tutelman PR, Langley CL, Parker JA, Cormier BT (2024). The prevalence of chronic pain in children and adolescents: a systematic review update and meta-analysis. Pain.

[16] Rosa BN, Noll M, Candotti CT, Loss JF (2022). Risk factors for back pain among southern Brazilian schoolchildren: a 6-year prospective cohort study. Int J Environ Res Public Health.

[17] Camargo EM (2020). Diretrizes da OMS para atividade física e comportamento sedentário: num piscar de olhos.

[18] Leite MN, Kamper SJ, O’Connell NE, Michaleff ZA, Fisher E, Silva PV (2023). Physical activity and education about physical activity for chronic musculoskeletal pain in children and adolescents. Cochrane Database Syst Rev.

[19] Sirajudeen MS, Alzhrani M, Alanazi A, Alqahtani M, Waly M, Manzar MD (2022). Prevalence of upper limb musculoskeletal disorders and their association with smartphone addiction and smartphone usage among university students in the Kingdom of Saudi Arabia during the COVID-19 Pandemic-A Cross-Sectional Study. Healthcare (Basel).

[20] Warda DG, Nwakibu U, Nourbakhsh A (2023). Neck and upper extremity musculoskeletal symptoms secondary to maladaptive postures caused by cell phones and backpacks in school-aged children and adolescents. Healthcare (Basel).

[21] Maayah MF, Nawasreh ZH, Gaowgzeh RAM, Neamatallah Z, Alfawaz SS, Alabasi UM (2023). Neck pain associated with smartphone usage among university students. PLoS One.

[22] Tsantili AR, Chrysikos D, Troupis T (2022). Text neck syndrome: disentangling a new epidemic. Acta Med Acad.

[23] Al-Hadidi F, Bsisu I, AlRyalat SA, Al-Zu'bi B, Bsisu R, Hamdan M (2019). Association between mobile phone use and neck pain in university students: a cross-sectional study using numeric rating scale for evaluation of neck pain. PLoS One.

[24] Hansraj KK (2014). Assessment of stresses in the cervical spine caused by posture and position of the head. Surg Technol Int.

[25] Ohayon M, Wickwire EM, Hirshkowitz M, Albert SM, Avidan A, Daly FJ (2017). National Sleep Foundation's sleep quality recommendations: first report. Sleep Health.

[26] Runge N, Ahmed I, Saueressig T, Perea J, Labie C, Mairesse O (2024). The bidirectional relationship between sleep problems and chronic musculoskeletal pain: a systematic review with meta-analysis. Pain.

[27] Bai Y, Kamarudin KM, Alli H (2024). A systematic review of research on sitting and working furniture ergonomics from 2012 to 2022: analysis of assessment approaches. Heliyon.

[28] Yamato TP, Maher CG, Traeger AC, Williams CM, Kamper SJ (2018). Do schoolbags cause back pain in children and adolescents? A systematic review. Br J Sports Med.

[29] Calvo-Muñoz I, Kovacs FM, Roqué M, Seco-Calvo J (2020). The association between the weight of schoolbags and low back pain among schoolchildren: a systematic review, meta-analysis and individual patient data meta-analysis. Eur J Pain.

